# Block versus Longitudinal Scheduling of Emergency Medicine Residents' Rotation in an Independent Children’s Hospital: Pediatric Emergency Medicine Attending Faculty's Perspective

**DOI:** 10.7759/cureus.6476

**Published:** 2019-12-27

**Authors:** Jennifer Mitzman, David P Way

**Affiliations:** 1 Emergency Medicine, The Ohio State University Wexner Medical Center, Columbus, USA; 2 Emergency Medicine, The Ohio State University, Columbus, USA

**Keywords:** pediatric emergency medicine, medical education, rotations, attending

## Abstract

Introduction

For many emergency medicine (EM) residency programs, pediatric education takes place at independent pediatric emergency departments (PEDs). Residency programs are charged with selecting a scheduling model for their residents' clinical experience at these PEDs. The main advantage of block scheduling is that it immerses the residents in pediatric care for a period of time and provides continuity of work with the same PED attending faculty. The longitudinal model offers residents continuous pediatrics experience throughout their training and allows them to treat illnesses related to the seasons (seasonal variation). The purpose of this project was to evaluate a shift from block to longitudinal scheduling through the eyes of the PED attending faculty members.

Methods

A questionnaire was designed by a committee to obtain attending faculty's opinions about resident scheduling, seasonality, and the factors they would consider to make decisions about resident autonomy in patient care. The questionnaire was reviewed by a survey expert and piloted; they were then delivered electronically to 60 faculty members at our affiliated children’s hospital emergency department (ED).

Results

The survey return rate was 63%. Most attending faculty expressed a preference for longitudinal over block scheduling because it eliminated the negative impact of seasonality on resident education. Others expressed positive features, including more sustained experience with pediatrics throughout training, and an experience that was more representative of day-to-day emergency medicine practice. A few attending faculty expressed concern that longitudinal scheduling would jeopardize attending faculty's familiarity with residents, making it difficult for residents to be entrusted to work autonomously. Most of the attending faculty suggested that their familiarity with a resident was a key factor in how they made decisions about the resident’s participation in procedures or resident autonomy; however, very few were concerned that longitudinal scheduling would impact their ability to “get to know a resident.”

Conclusion

Attending faculty mostly thought that longitudinal scheduling was better than block scheduling. While they acknowledged that their familiarity with the resident was the driver of faculty entrustment in the PED, they did not express any concern that the scheduling change would affect their ability to get to know the residents. Other solutions, including a mixed scheduling model to address both issues, are also proposed.

## Introduction

Emergency medicine (EM) residency programs are tasked with educating residents to care for patients of all ages, including pediatric patients (those under the age of 18). Regarding pediatric experiences for EM residents, the Accreditation Council for Graduate Medical Education (ACGME) requires four months of critical care of infants and children, and five months or “20 percent of all emergency department encounters dedicated to the care of patients younger than 18 years of age [1].” Additionally, residents are required to demonstrate proficiency in procedures most commonly seen in the pediatric emergency departments (PEDs), such as laceration repair [1]. They must also demonstrate competency in “pediatric medical and trauma resuscitation” [1]. Most importantly, the ACGME asserts that residents must “undertake progressive levels of responsibility,” by “assuming roles which permit them to exercise patient care skills with greater independence [1].” This means that programs must evaluate and track a resident’s progress throughout their training.

To meet pediatric accreditation requirements, EM residency programs are tasked with organizing resident experiences in clinical settings that involve the care of children. For many programs, this requires partnering with independent pediatric care facilities. The most recent information, from a 1996 Society of Academic Emergency Medicine pediatric emergency medicine (PEM) training task force, suggested that as many as 85% of EM residencies were reliant on PEM rotations at tertiary pediatric hospitals, designated PEDs, and exclusive children’s hospital emergency departments (EDs) for the pediatric education of their residents [2].

While offering high-quality educational experiences, EM residents’ participation in PEDs separate from their home institution poses unique challenges for residency programs. The most significant of these being unfamiliarity between residents and their supervising PED attending faculty stemming from limited interactions [2]. The unfamiliarity of the resident with the attending faculty complicates the tracking of progressive levels of responsibility mandated by the ACGME [1]. 

An additional challenge involves seasonal variation, a characteristic of pediatric visits to the ED [3-4]. Because children commonly suffer some emergent or urgent afflictions during certain seasons of the year, programs must strategically schedule EM residents so that they are able to gain experience treating those pediatric conditions related to the seasons.

Given these challenges, EM residency programs are often tasked with organizing the participation of their residents in the independent children’s hospital PEDs to meet their educational needs. Most EM residency programs maintain a block scheduling structure, the practice of sending an EM resident to the PED for stretches of time, usually for a month, during which time they do not see any adult patients. An alternative is longitudinal scheduling, which refers to the assignment of residents to shifts in the PED throughout the year, interspersed with other educational experiences. Those who advocate for block scheduling tend to highlight the advantages of immersing the resident in pediatric care and the continuity of working with the same PEM attendings. Those who advocate for the longitudinal model argue that it gives residents more experience with illnesses related to the seasons (seasonal variation) [5-6]. What is unclear are the trade-offs involved in selecting one model over the other.

The purpose of this project was to evaluate Ohio State University's recent transition from block scheduling to longitudinal scheduling from the perspective of one of our most important stakeholders, the pediatric attending faculty. Specifically, we wanted to determine what faculty perceived to be the advantages and disadvantages of block versus longitudinal scheduling for addressing the dynamics of both seasonality and residents' educational development over time. 

## Materials and methods

Population and setting

The study was conducted at an urban tertiary care PED in the fourth largest free-standing children’s hospital in the US (Nationwide Children’s Hospital, Columbus, OH), with an annual census of approximately 90,000 pediatric patients [7]. The PED provides educational rotations for over 300 residents from 17 different programs annually. Among the specialties represented by these residency programs are family medicine, pediatrics, radiology, anesthesiology, combined internal medicine-pediatrics, and five separate EM residency programs. The Ohio State University's three-year residency program is the largest contributor to EM residents to this PED, comprising 56 residents or 18.7% of the total number of residents who receive their pediatric education through this setting. Prior to the academic year 2016-17, our residents were scheduled in block rotations that lasted one month. They completed one-and-a-half blocks in their first year (with a half month during orientation), and two month-long blocks in each of the years two and three. This study was initiated at the end of the academic year 2016-17 at which time our program transitioned from block rotations to a longitudinal, integrated, year-round shift schedule. The Human Subjects Review Board at Nationwide Children’s Hospital, Columbus, OH (IRB18-00838) determined that this study was exempt from their review.

Measurement instruments

We developed a questionnaire to ask PEM attending faculty about how they thought the changes from block to longitudinal scheduling would impact the PEM education of EM residents. The questionnaire included questions about seasonal variation, the value of working with the same resident over time, and entrustment decisions. Questionnaires were originally drafted by a panel comprising PED faculty, educators who belong to our EM residency program’s curriculum committee, and an EM resident, and then evaluated by a psychometrician with expertise in survey design. The end product was reviewed and piloted by EM residency program administrators.

Initially, we asked PEM attending faculty whether they thought that seasonal variation was an important consideration for organizing the education of EM residents. They were also asked to explain their position and provide suggestions as to the best practices to limit the negative impacts of seasonal variation. The second part of the questionnaire used open-ended questions to determine how the attending faculty made decisions regarding the participation of the EM resident in pediatric patient care. These included decisions to allow an EM resident to 1) see a patient, 2) perform procedures, and 3) manage cases on their own. Finally, we attempted to have respondents verify factors related to how they made these entrustment decisions by selecting factors from a list (see Appendix A) [8-11].

Questionnaires were distributed through Qualtrics during the months of June and July 2017 (Qualtrics, June 2017; Provo, UT). We used the Dillman Tailored Design Method to guide our survey implementation, which included a preliminary notice about the study, dissemination of the questionnaire, a follow-up reminder, and a second round of questionnaire dissemination [12]. Access to the questionnaire was closed after eight weeks. 

Data analysis

Checklist items were presented as descriptive statistics (counts and frequencies). Attending responses to open-ended questions were independently coded by two authors (JM and DPW). For questions regarding the attending faculty's decision as to how the resident could participate in the PED service, we used the four factors that influenced entrustment decisions from the literature as preliminary categories [8-10, 13-14]. The two coders independently sorted responses into these preliminary categories based on synonymous language for each question separately and then discussed each other’s coding to reconcile differences. For the items related to seasonality and the switch to longitudinal scheduling, the coders used open coding for the first round, then more focused coding during the second and third rounds. Again, discrepancies were discussed and resolved at the end of each round.

## Results

The return rate for PEM faculty was 63.3% (38 of 60), with eight questionnaires being partially completed. Table 1 shows the demographic profile of survey respondents as compared to non-respondents. Non-significant statistical test results show that the respondent and non-respondents were similar across all four demographic characteristics collected. The average years of experience in practice (years out of residency training) were almost 14 years for the respondents versus close to 10 years for non-respondents. Gender representation was consistent with the population of attending physicians who work in our PED, (71-29% split between women and men). Most of the respondents (92%; 35 of 38) had completed pediatric rather than EM residency programs. Almost two-thirds of the respondents were boarded in PEM, while 37% were boarded in pediatrics only. Table 1 below lays out counts and percentages of survey respondents vs. non-respondents for 60 pediatric emergency medicine attending faculty by personal demographics: gender, residency training background, board certification, and years out of residency. Chi-square of proportion and t-tests report as to whether the respondents are significantly different from the non-respondents with regard to demographic profile.

**Table 1:** Demographics of survey respondents vs non-respondents EM: emergency medicine; PEM: pediatric emergency medicine

Characteristic	Respondents	Non-respondents	Total
Gender			
Female	27 (66%)	14 (34%)	41 (68%)
Male	1 (58%)	8 (42%)	19 (32%)
	X^2 ^= 0.34, df = 1, p = 0.58		
Training background			
Pediatrics	35 (61%)	22 (39%)	57 (95%)
Emergency medicine	3 (100%)	0 (0%)	3 (5%)
	X^2 ^= 1.83, df = 1, p = 0.29		
Board certification			
Pediatrics only	14 (54%)	12 (46%)	26 (43%)
Pediatrics & PEM	21 (68%)	10 (32%)	31 (52%)
EM & PEM	3 (100%)	0 (0%)	3 (5%)
	X^2 ^= 3.00, df = 2, p = 0.22		
Experience, years			
Mean	13.7	9.8	12.3
Standard deviation	9.0	8.6	9.0
	t= 1.65, df=58, p= .11		
Total	38 (63%)	22 (37%)	60 (100%

Longitudinal Scheduling

When asked whether the shift to longitudinal scheduling was a positive step in improving resident education, 76% responded yes. Most of these respondents (76%) said that eliminating the negative impact of seasonal variation was the primary reason. Other reasons offered included eliminating long gaps of time in which the resident would not see pediatric patients and providing an experience that was more representative of day-to-day ED practice.

Only two dissenting voices suggested that the longitudinal scheduling posed more problems than it solved. Both believed that longitudinal scheduling would reduce “faculty familiarity” with the resident, which would lead to reduced resident autonomy and opportunities to perform procedures. This sentiment was best captured by the following comment:

“Getting to know a resident is highly important to help them learn and to feel comfortable with progressive delegation. Having a few shifts in a row helps. Spreading out the experience may delay or inhibit getting to know the resident. Overall, my calculation is that knowing and developing relationships is highly important for effective training and tracking of progress. Given the fragmentation of our schedules, I see the longitudinal experience as a net negative to neutral from that perspective.” 

The final open-ended question about concerns or questions regarding longitudinal scheduling resulted in several salient themes: 1) residents need a block of time to learn the system in an unfamiliar hospital, and longitudinal scheduling would prevent them from learning and settling into the children’s hospital system; 2) getting to know the resident will take longer under the longitudinal scheduling model and make it more difficult to evaluate their educational growth over time. Without knowing them well, the resident is also not likely to receive the autonomy they need.

Seasonal Variation

Attending faculty were nearly unanimous (97.4%, 37 of 38) in their belief that seasonal variation impacted the education of EM residents; however, there was a diversity of opinion about whether the impact was positive or negative, and what to do to limit the negative impact. Those who said that seasonal variation had a positive impact (26%) explained that seasonal variation emulated “real world” medicine, and seeing pediatric patients across different seasons helped the learner to better understand and anticipate the underlying seasonal case-mix patterns.

Attending faculty who perceived seasonal variation negatively (13%) referred to the limits of block scheduling and remarked that some residents never got to see certain disease processes that are not “in season” when they rotated through the PED. The other 61% cited both positive and negative aspects of seasonal variation on PEM education. These faculty suggested that if seasonality was considered when scheduling, for example, by ensuring that blocks were distributed throughout the year, then it provided the resident with adequate experience. However, if not considered during scheduling, seasonality would result in learning deficits.

When asked to provide best practices to control for the negative effects of seasonality, faculty generally suggested some form of strategic scheduling. Of the 26 who provided specific suggestions, 11 (42%) endorsed longitudinal scheduling, nine (35%) endorsed strategic block scheduling so that the resident had at least one month in each season (summer, fall, and winter), and two suggested a mix of block and longitudinal scheduling. The remaining four suggested that it didn’t matter which scheduling method was used as long as residents were scheduled for different seasons.

Entrustment 

In response to the open-ended question about allowing residents to see patients, nearly three-fourths (71%) of the attending faculty said that they were not involved in this decision and that residents were guided by department algorithms. Attending faculty reported that this decision was a matter of logistics rather than trust, as residents followed the protocol of seeing the next sickest patient with the next longest wait time. Yet when asked to check off the factors involved, more than two-thirds checked “familiarity with the resident and resident’s ability” as factors related to this decision. Additionally, more than 60% also checked training level and patient acuity (see Table 2). 

**Table 2:** PEM faculty responses to questions about factors that faculty employ to regulate the participation of an EM resident in patient care during rotations in a PED EM: emergency medicine; PGY: postgraduate year; PEM: pediatric emergency medicine; PED: pediatric emergency department

	Decision to see patients, n (%)	Decision to allow procedures, n (%)	Decision to offer autonomy, n (%)
Patient-related factors			
Patient acuity	19 (61.3)		29 (93.5)
Procedural difficulty		25 (83.3)	
Parent consent	0 (0)	3 (9.7)	1 (3.2)
Resident-related factors			
Attending assessment of the resident’s ability	22 (71.0)	28 (90.3)	30 (96.8)
Resident’s level of training (PGY)	19 (61.3)	25 (80.6)	29 (93.5)
Resident’s confidence	13 (41.9)	23 (74.2)	21 (67.7)
Resident’s personality	6 (19.4)	8 (26.7)	14 (45.2)
Resident’s program affiliation	11 (35.5)	7 (23.3)	10 (32.3)
Environmental related factors			
Presence of an EM Fellow	16 (51.6)	20 (66.7)	14 (45.2)
Conditions of the department (flow)	1 (3.2)	4 (13)	2 (6.5)
Competition between learners	0 (0)	8 (25.8)	0 (0.0)
Faculty-related factors			
How well attending faculty knows the resident	21 (67.7)	23 (74.2)	31 (100)
Resident’s education needs as determined by the faculty	3 (9.7)	3 (9.7)	0 (0.0)

Attending faculty's responses to the open-ended questions related to factors that impacted their decision to allow residents to perform procedures clustered into two themes: the resident’s level of training and whether they had performed the procedure before. However, their checklist responses included resident ability, procedural difficulty, and how well the attending knew the resident. Two-thirds of the faculty also said that the presence of a PEM fellow was a factor. Supplementary comments implied that the PEM fellow was considered a potential educational competitor, whose needs for the procedural experience took precedence over the needs of the resident. 

When asked about factors related to resident autonomy, more than 75% of the faculty said this decision was based primarily on their familiarity with the resident. This response was confirmed when 100% of the faculty checked familiarity as an important factor. Patient acuity, level of training, and resident ability were also identified by more than 90% of the faculty respondents as key factors.

## Discussion

The PED attending faculty provided some key insights as to how our move from block to longitudinal scheduling affected our residents' pediatric education. They identified a number of advantages associated with longitudinal scheduling. First, they felt that this model of scheduling was the best way for residents to see all illnesses and injuries related to seasonality. They also suggested that interspersing individual shifts between pediatric and adult EDs would more closely resemble the most common care setting, in which adults and patients are seen within the same space. Finally, participants suggested that spreading out their pediatric education longitudinally throughout their entire training would give residents more sustained contact with pediatric patients throughout residency, giving them a stronger foundation of knowledge and skills related to the care of children.

The disadvantages identified by our PEM attending faculty were that pediatric knowledge and skills would take longer to acquire, and would be hampered by the length of time it took the new residents to adapt to working in a new setting. Learning the flow and practice patterns of a new PED, including order sets and electronic health record systems, took longer when residents were only in the PED site for one shift every one or two weeks. The last disadvantage identified by PEM attending faculty involved the establishment of working relationships. Attending faculty suggested that shift scheduling might make it more difficult for attendings to become sufficiently familiar with EM residents in order to entrust them with patient-care responsibilities. This hit-or-miss approach to scheduling also prevented residents from spending sufficient amounts of time with any one attending faculty member so that they were familiar enough to evaluate and track the resident’s educational progress. Additional research is needed on the best methods for promoting attending faculty-resident relationships that lead to progressive entrustment and autonomy of EM residents in the pediatric ED. 

We know from the work of others that “familiarity” is a necessary but not sufficient condition for “entrustment” [15-16]. Consequently, we had concerns that the change in scheduling would directly impact the attending’s opportunity to “get to know” the EM residents and their educational achievement. What was interesting but a little concerning was that attending faculty identified familiarity with a resident as a key to how they supervised the EM resident, but did not recognize that familiarity might be harder to achieve with longitudinal scheduling. 

Future research is needed to more directly document the encounters the residents experience in the PED. We are in the process of building a patient-encounter database obtained from electronic medical records to inform us about the patient acuity levels that our residents experience and the procedures they perform in the PED. This will also allow us to see whether the change from block scheduling to longitudinal scheduling has a negative impact on our resident’s pediatric education and may offer clues to better scheduling models. In the interim, we recommend that EM residencies employ a mixed scheduling design when confronted with coordinating their residents at independent PEDs; we also recommend to include a block of time during the first year to immerse their interns in a pediatric care environment, followed by longitudinal scheduling in the remaining training years, interweaving pediatric shifts into the resident’s regular schedule on a weekly or biweekly basis.

An alternative to mixed scheduling to address the lack of attending faculty's familiarity with the resident under the longitudinal scheduling model is to match residents with a core group of attending faculty and schedule them to work together on shifts over the residents' training. This model of scheduling was demonstrated to be a desirable alternative for medical students on emergency medicine clerkships by Bernard et al. [17]. 

A final alternative is to place the responsibility for tracking educational progress through the implementation of a passbook/passport system as proposed by TE Read [18]. Under this system, the resident would seek out required chief complaints or procedures and log them over the course of their shifts in the PED. The logs would be reviewed on a routine basis by the program leadership to evaluate the resident’s educational progress over time.

Limitations

The limitations inherent to projects involving questionnaires is the self-reporting nature of the data gathered from attending faculty. While we were interested primarily in the perspective of PEM attendings, we recognize that they may not be completely aware of the factors that go into their decisions about resident involvement in patient care.

We specifically identified Ohio State University Emergency Medicine as the sponsors of this research; however, since our PED attending faculty work with residents from 17 different residency programs, the possibility exists that PED faculty program affinities and training backgrounds may have impacted return rates and survey responses. 

Finally, because this study was conducted in one pediatric site to answer questions about scheduling for one EM residency program, the results are somewhat limited with regard to generalizability to other sites. However, we believe the issue regarding how to best integrate EM residents into a separate pediatric care site for some of all of their pediatric education is a common problem that is faced by many programs.

## Conclusions

PED attending faculty effectively weighed the advantages and disadvantages of longitudinal scheduling and provided a model for the future. They also identified and confirmed that the attending faculty's familiarity with the resident, especially concerning their ability, their experience, and their level of training, was a key factor that influenced how they made decisions regarding the resident’s involvement with patient care in the PED. However, the faculty did not perceive longitudinal scheduling as an impediment to becoming sufficiently familiar with EM residents so that they would gain progressive levels of responsibility in the PED.

## Appendices

Pediatric Emergency Medicine Attending Questionnaire

Dear Pediatric Emergency Medicine Faculty:

We are writing to ask you to take ten minutes to complete a brief survey about your interaction with emergency medicine residents in our department.  Our hope is to catalogue the collective opinion of faculty to determine whether a longitudinal pediatric curriculum for residents might improve their educational experience. We plan to disseminate our survey results over the next year.

We are tracking surveys as they are returned so that we do not have to trouble you further once you respond. However, individual responses will be kept strictly confidential and will only be known to the data analyst. Results will only be reported in aggregate form protecting the identity of individual respondents.  Please note that you may skip questions that you do not wish to answer or withdraw your participation at any time.

Please complete the survey by Friday, August 4, 2017

We greatly appreciate your contribution to this project. If you have further questions about the nature or results of this study or if you feel you have been harmed as a result of participation, please feel free to contact us.

With sincere gratitude,

Signatures:

The Ohio State University Emergency Medicine Residency Program Director

The Division Chief of Emergency Services at Nationwide Children’s Hospital

The Primary Investigator for this study                    

Prompt: Seasonal variation in case mix at pediatric emergency departments is thought to have an impact on the educational experience of emergency medicine residents.

1. Do you believe seasonal variation has had an impact on the education of EM residents?

    YES    -> go to Item 2

     NO      -> go to Item 5

2. What type of impact does seasonal variation have on education?

    POSITIVE      -> go to Item 3

    NEGATIVE   -> go to Item 3

    BOTH             -> go to Item 3

   NEITHER       -> go to Item 5

3. How does seasonal variation impact resident education?

4. Are there changes we should make to resident education to minimize the impact of seasonal variation on resident education?

    -> go to Item 6

5. Why doesn’t seasonal variation have an impact on resident education?

Prompt: The following questions ask about how you make decisions about a resident’s participation in the Peds ED.

6. What factors go into your decision to let an EM resident see a patient?

7. What factors go into your decision to let an EM resident perform a procedure on a patient?

8. With regard to patient care, what factors go into your decision about how much autonomy you offer an EM resident?

Prompt:  The following items ask about specific factors from the literature that may guide your decisions about how a resident is permitted to interact with patients. Please answer true or false for each factor.

9. This factor plays a role in my decision to allow a resident to see a patient.

·      Patient acuity [True vs. False]

·      My informal assessment of resident’s ability

·      Resident level of training

·      Presence of an EM Fellow

·      Resident’s program affiliation

·      How well I know the resident

·      Resident’s confidence

·      Resident’s personality

·      Other….please list

10. This factor plays a role in my decisions about resident autonomy. 

·      Patient acuity

·      My informal assessment of resident’s ability

·      Resident level of training

·      Presence of an EM Fellow

·      Resident’s program affiliation

·      How well I know the resident

·      Resident’s confidence

·      Resident’s personality

·      Other….please list

11. This factor plays a role in my decision to allow a resident to perform procedures on a patient.  

·       Procedural difficulty

·       My informal assessment of resident’s ability

·       Resident level of training

·       Presence of an EM Fellow

·       Resident’s program affiliation

·       How well I know the resident

·       Resident’s confidence

·       Resident’s personality

·       Other….please list

Prompt: The OSU Emergency Medicine residency program has moved to longitudinal scheduling of their residents in the Pediatric Emergency Department. This means that EM residents are now rotating in the pediatric emergency department on shifts throughout the year instead of month-long blocks during their residency.  

12. Do you think that this is a positive step to improve resident education?    

     YES -> go to Item 13

      NO -> go to Item 14

13. How do you think the longitudinal pediatric experience will improve resident education?

14. Why don’t you think this is a positive step?

15. Please share any concerns or questions you have about the longitudinal pediatric experience. 
